# Assessment of soil quality in arid zones using principal component analysis and GIS-based modeling

**DOI:** 10.1371/journal.pone.0337063

**Published:** 2025-12-02

**Authors:** Mohamed S. Shokr, Mohamed E. M. Jalhoum, Ahmed S. A. Sayed, Mohammed Saeed, Nazih Y. Rebouh, Elsayed Said Mohamed, Ibraheem A. H. Yousif, Hend H. Abdelhameed

**Affiliations:** 1 Soil and Water Department, Faculty of Agriculture, Tanta University, Tanta, Egypt; 2 National Authority for Remote Sensing and Space Sciences, Cairo, Egypt; 3 Pedology Department, Water Resources and Desert Soils Division, Desert Research Center, Cairo, Egypt; 4 Soils and Water Department, Faculty of Agriculture, Al-Azhar University, Cairo, Egypt; 5 Institute of Environmental Engineering, RUDN University, Moscow, Russia; 6 Soil Science Department, Faculty of Agriculture, Cairo University, Giza, Egypt; 7 Soil and Water Department, Faculty of Environmental Agricultural Science, Arish University, Arish, Egypt; NGRI: National Geophysical Research Institute CSIR, INDIA

## Abstract

**Background:**

Assessing the quality of the soil is a crucial first step in agricultural management. A major obstacle to raising agricultural output, economic growth, and environmental health has been the decline in soil quality. One of the most often used metrics for evaluating soil quality is the soil quality index (SQI), which is frequently calculated using principal component analysis (PCA).

**Methodology:**

In this study, a soil quality index in the southwest of the Ismailia Governorate, Egypt, was evaluated and mapped using a geographical information system (GIS) and multivariate analysis. (PCA). Fifty- one soil samples were gathered for this purpose, and they were examined using established procedures. The dataset was broken down into new variables using principal component analysis (PCA) to avoid multi-collinearity. Relative weights (Wi) and soil indicators (Si) were then established and used to calculate SQI. The SQI comprises three quality zones.

**Results and discussion:**

The first zone has a very good quality index, accounting for about 65.66 (ha) of the entire area. Soils in this zone were defined by low salinity of the groundwater and adequate values of each soil attribute. The second zone, which makes up about 414.76 ha (67.5%) of the total area, is characterized by its good-quality soil. About 133.91 ha (21.8%) of the total land area is in the third zone, which is fair (bad quality). Low concentrations of soil organic matter (SOM), salinity, accessible nitrogen (N), phosphorus (P), potassium (K), and cation exchange capacity (CEC) had the greatest effects on the SQI of the studied location. Combining PCA and GIS enables a precise and efficient evaluation of the SQI.

**Conclusion:**

Decision-makers can identify regions with very good, good, and poor soil quality by examining the generated spatial distribution maps. Additionally, they can learn how each characteristic influences plant growth. In addition, The methodology outlined in this work can be readily replicated in similar situations in arid regions, enabling local authorities and decision-makers to make use of the quantitative results achieved to guarantee long-term development.

## 1. Introduction

The issue of food insecurity is currently one of humanity’s most pressing concerns. Global food consumption is predicted to climb by 70% by 2050, and agricultural productivity will play a critical role in securing global food security [1]. By the year 2000, there were 6.1 billion people on the planet, up from about 250 million in the year 1000. By 2050, that number is expected to rise to 9.8 billion. Unsustainable soil deterioration is already a result of the current intensification of agricultural operations [2]. The main manifestations of ecological degradation include the loss of organic matter and greenhouse gas emissions, excessive fertilizer use, erosion, pollution, acidification, salinization, and genetic diversity loss. Continuous soil degradation is harming the ecosystem and reducing the long-term capacity of soils to offer services to humans, such as future food production [2]. Advancing the Sustainable Development Goals (SDGs) relies heavily on sustainable soil management [3]. Numerous SDGs are directly influenced by soil characteristics and processes, as well as by their sustainable management. Of particular importance are SDDG #2, 3, 6, 13, 15, and 17. Moreover, soil quality and its management have indirect impacts on several SDGs (e.g., 1, 5, 6, 8, 10, and 16) [3].

It is therefore anticipated that there may be a shortage of land and agricultural resources. Long-term assessment is necessary to meet this demand [4]. Soil is one of the most crucial resources for agriculture and livestock, which are key to fulfilling the global demand for food security [5]. Soil quality refers to the capacity of a specific soil type to operate within the limits of a natural or managed ecosystem to sustain plant and animal productivity, maintain or improve water and air quality, and support human health and living conditions [6]. A trustworthy evaluation requires a precise, multidimensional quantification of soil quality to facilitate sustainable soil management [7]. However, the computation of the soil quality index (SQI) often requires the use of numerous variables to make more accurate decisions, which adds to the task’s complexity and, on occasion, its cost [8]. The physical, chemical, and biological characteristics of the soil can be evaluated to calculate the SQI. It is common to receive redundant information while using large datasets. As a result, principal component analysis (PCA) is a multivariate technique that enables dataset reduction, hence enabling the determination of management objectives [9]. According to Ghaemi [10] soil quality indicators are quantifiable characteristics of soil that show the responsiveness of soil productivity or soil-environment functionality. These indicators are used to determine whether soil quality is increasing, staying the same, or decreasing. The most popular techniques for assessing soil quality are thought to be soil quality indices because of their simplicity, adaptability, and quantifiability [11]. According to Singh and Khera [12], these indices show the combined effects of several soil characteristics, including physical, chemical, and ecological ones, as well as the contribution of each indicator to soil quality. They are used to choose and combine soil quality indicators into a single index, and act as a management tool to give land managers all the data they need to make decisions about the management of agroecosystems [13].

Salinity, cation exchange capacity, organic matter content, pH, phosphorus availability, nutrient cycling, and the number of contaminants in the soil are chemical indicators, whereas soil depth, bulk density, porosity, aggregate stability, and texture are physical indicators that can be used to quantitatively assess the fertility state and general health of the soil [14,15]. In the upcoming decades, there will be a pressing need to preserve and enhance the quality of agricultural soils [13]. The most popular approach for calculating SQI is principal component analysis (PCA), which occasionally falls short of accurately differentiating soil quality. Olive [16] states that the dispersion structure is described by principal component analysis along with a few linear combinations of the original variables. When fewer variables are available for different groups of people, PCA can analyze multivariate relationships and explain data variation [8]. PCA finds a small number of linear combinations (principal components) of a set of variables to preserve as much information about the original variables as feasible [16,17]. Prior research on the Upper Tigris Basin [18], Iran [19], Farafra Oasis, Nile Delta, and Beni-Suef, Egypt [20–22], has proven the effectiveness of PCA in establishing critical variables for dryland agroecosystems. Not all of the indicators used to evaluate land resources are equally useful for figuring out how ecosystems work. As a result, it is necessary to assess each parameter’s proportional importance (weight) [23]. In research about to the evaluation of natural resources, the PCA has also been employed as a multi-indicator weighting approach [24,25]. Regardless of the kind of soil, PCA and common soil parameters approaches provided consistently comparable results and might be considered helpful tools to support soil management initiatives [26]. Geographic Information System (GIS) and (Remote sensing (RS) are two methods that can be combined to improve one another [27]. RS is a quicker and less expensive method of gathering a lot of information about soil and topography characteristics, as well as land under different land use/land cover types, than thorough field surveys [28]. These types of data can be stored, manipulated, and arranged using GIS into themes or subjects that reflect the various facets of intricate environmental issues [29]. Spatial modelling can incorporate spatially referenced datasets through the combined use of RS and GIS [30].

Soil property characterization, modeling, and mapping at different geographical and temporal scales are required for SQI study using GIS technique [31]. Therefore, mapping and identifying the spatial variation of soil properties in hitherto unexplored locations may benefit from the combination of PCA analysis and GIS. GIS technology has made it easier to calculate the spatial variability of several phenomena, including soil property research [32]. Therefore, evaluating the spatial variation of soil parameters and forecasting them in unsampled places can benefit from the use of combined GIS and geostatistical analysis. Among the most popular interpolation techniques is kriging [33,34]. Ordinary kriging (OK) serves as a fundamental application of this method, yielding a prediction that is both optimal and free from bias [35]. OK simulates spatial variability through various variograms that can reduce the variance of prediction inaccuracies and provide different map outputs [36]. Mustafa [8] demonstrated how GIS technology has made it possible to compute the spatial variability of several phenomena, including soil property research. Thus, the combination of GIS and geostatistical analysis may be very beneficial for assessing the spatial variance of soil parameters and those expected in un-sampled sites [15]. In addition, statistical methods like PCA and GIS geostatistical approaches work well and are simple to use in areas with limited data availability [37]. This approach can adequately enable precise farming since it is predicated on the discovery of homogenous subsets of comparable yield-limiting parameters [38]. The primary goal of the current study is to evaluate, describe, and map the SQI by a multivariate analysis that combines PCA and GIS. Using PCA and GIS approaches together would create new avenues for carrying out more accurate and thorough simulations of the dryland agroecosystems services.

## 2. Materials and methods

### 2.1. Study area

**Fig 1:**
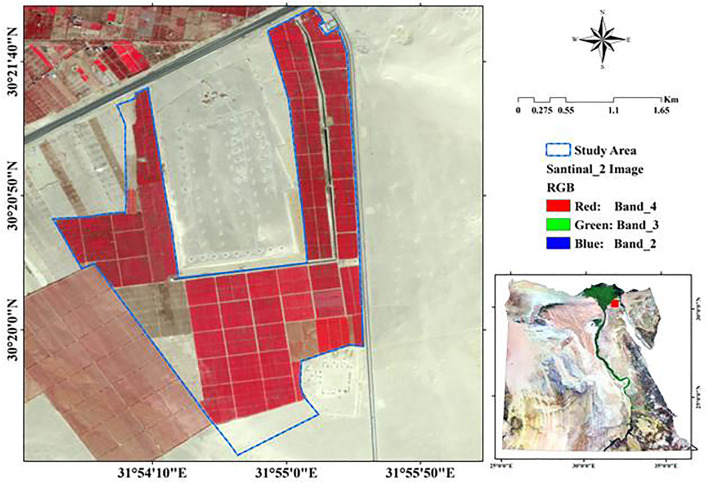
Location Map of study area.

The study area is located southwest of El- Ismailia Governorate, Egypt, and spans an area of 614.33 hectares(ha) between the latitudes of 30◦ 19′ 12.88′′ and 30◦ 22′ 0.02′′ N and the longitudes of 31◦ 53′ 33.76′′ and 31◦ 55′ 29.47′′ E (Fig 1). The satellite image was downloaded from (www.esa.int/Applications/Observing_the_Earth/Copernicus/Sentinel-2 in July 2024). The governorate is strategically located as the center-governorate for the Suez Canal area and serves as a logistical hub at the intersections of the roads leading to Cairo, Suez, Port-Said, and Sinai. As a hub for international trade and shipping, the Egyptian government has initiatives to grow the Suez Canal industry [39]. The area is considered promising for most agricultural crops due to the depth of the soil and the provision of the main agricultural requirements [40]. Thus, this study is regarded as a first step toward the development of sustainable agriculture in the area under investigation. The area is located in a prime location and very close to the main roads. The study was conducted on publicly accessible property and did not involve interactions with controlled species or habitats, so field site access and research activities did not require special permits. The area is considered promising for most crops due to the depth of the soil and the provision of the main agricultural requirements. The average temperature is 22 °C, with a mean annual maximum of 29.8 °C. The yearly rainfall is limited to about 25 mm, which results in minimal precipitation [40]. The typical relative humidity range is between 38 and 63%. There is a Torric soil moisture regime, and the study area is quite hot. The region under investigation is distinguished by a long, dry summer with high temperatures and rates of evaporation, followed by a short, moderate winter with infrequent autumnal rainfall. There are three types of Ismailia climate: a long period of chilly winter, an intermediate season with light rainfall, and a moderate summer with 75% humidity [41]. These are typically dominated by mild weather throughout the year. Only 50 mm of rain falls there annually, mostly during the winter months; as a result, the region experiences droughts due to a combination of low rainfall and an increase in evaporation rates [41]. The region under study is entirely covered by Quaternary silt deposits, which may conceal past tectonic deformations [42]. Southward-facing Miocene bedrock exposures are made up of limestone, clay, and sandstone. The shallow and deeper aquifers are the two hydrogeological units that make up the region surrounding the Ismailia Canal. While the shallow aquifer’s groundwater salinity ranges from 340 to 7650 mg/L, the deeper aquifer’s groundwater salinity is modest and seldom surpasses 1500 mg/L [43].

**Fig 2:**
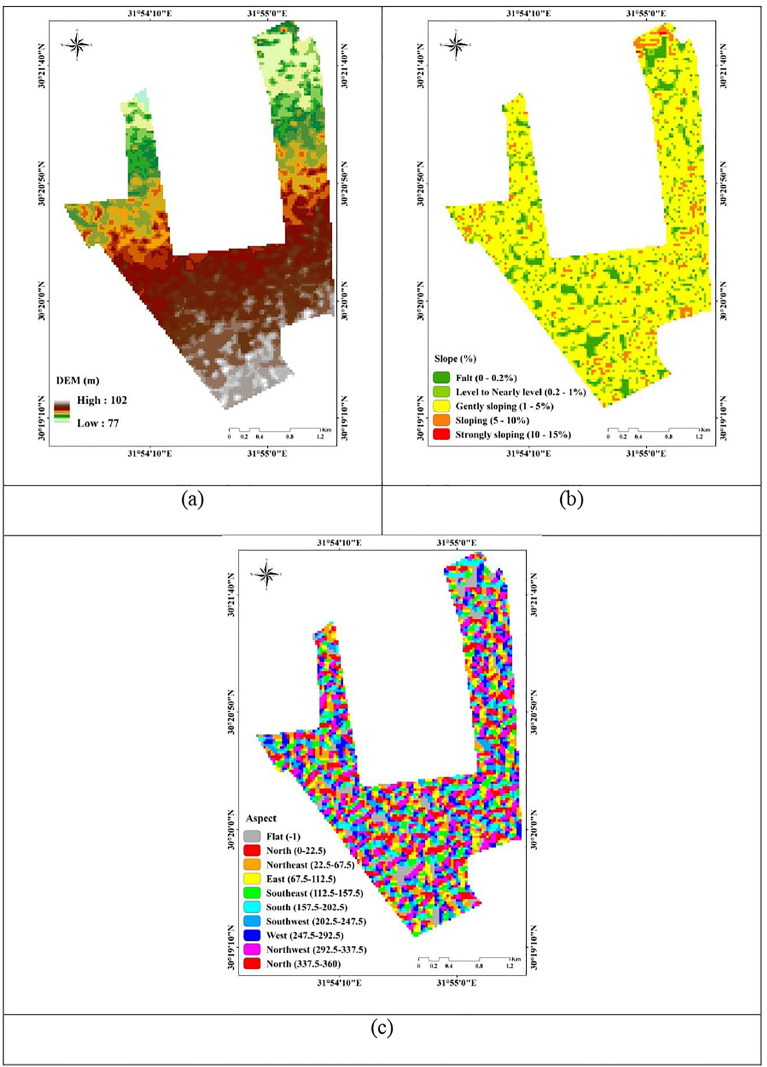
Land surface characteristics of the study area. (a) Digital Elevation Model (DEM), (b) slope, and (c) aspect.

With a spatial resolution of 30 m, the DEM was acquired by the NASA Shuttle Radar Topographic Mission (SRTM) (https://earthexplorer.usgs.gov accessed on 10 Jul 2024). The topographical analysis of the study region (Fig 2) revealed that the area surface elevation varies between 77 and 102 meters above sea level (m.a.s.l). While the predominant slope is gently sloping (1–5%). The derived aspect used in the study area ranges between (−1) flat to north (360).

### 2.2. Sampling and soil analysis

**Fig 3:**
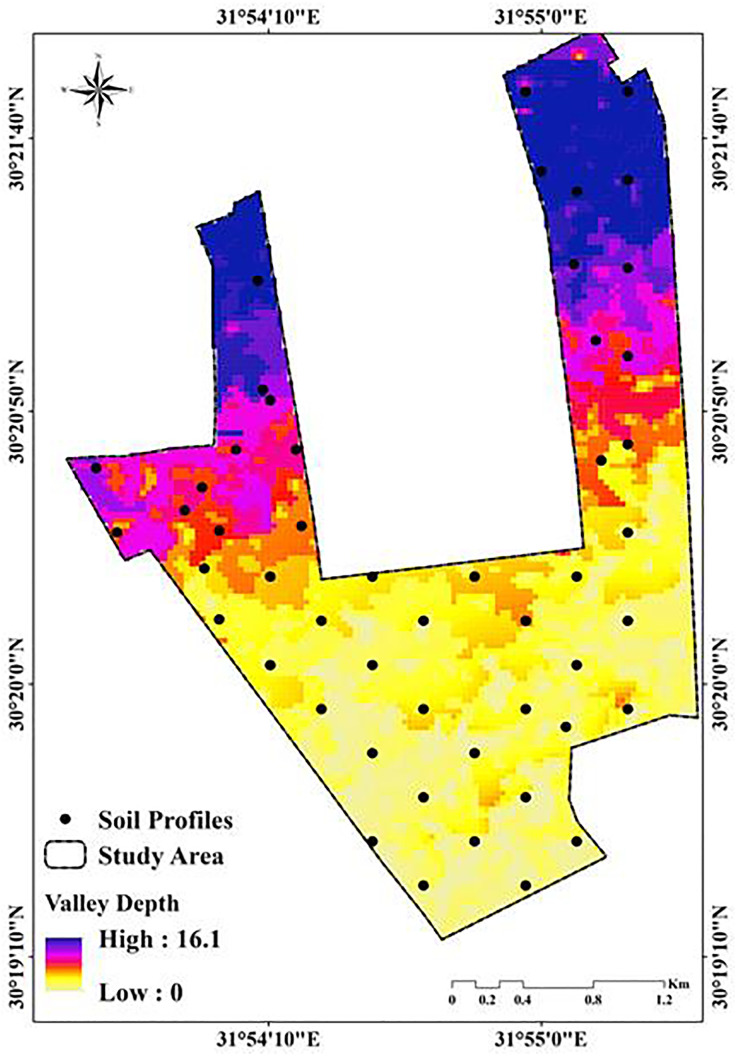
Spatial distribution of soil samples within the study area.

Fifty-one soil samples, ranging in depth from 0 to 60, were gathered from various locations, using a Garmin GPS, the precise positions of the soil samples were located in the field and displayed on a map (Fig 3). From each site, a single mixed sample that represented the root zone soil was collected. Within the research area, which is characterized by a wide range of physiographic features, the selected places represent spatial differences. Each soil sample was collected using a grid method at 350 x 350 m^2^ interval sto ensues representing of soil properties and geomorphological units of the study area. As explained by Van Reeuwijk [44], and USDA [45], to get ready for physical and chemical testing, the samples were air-dried, ground into a powder, and then sieved through a 2 mm screen. Using a glass electrode pH meter, the soil reaction (pH) values in a soil: water (1:2.5) suspension were ascertained. The volumetric measurement of calcium carbonate content was done with Collin’s Calcimeter [46,47]. The soil’s electrical conductivity was measured in saturated soil paste extract (EC) [48]. A flame-photometer device was used to quantify the amount of potassium that was available after it was extracted using 1N NH4OAc at pH 7 [49], The micro-Kjeldahl Method was used to distill available nitrogen; the ascorbic acid method was used to determine available phosphorus calorimetrically; mathematical equation was used to compute the exchangeable sodium percentage (ESP) by Van Reeuwijk, Bashour and Sayegh [44,50]. CEC was measured using the sodium acetate method, and the soil organic matter was ascertained using the Walker and Black method [48].

### 2.3. Statistical analysis

One exploratory, multivariate statistical method for analyzing data variability is principal components analysis (PCA). It is commonly used in environmental data, where complicated interrelationships between variables are challenging to find and visualize, and datasets may be sizable and challenging to comprehend. Compared to univariate procedures, which can lead to errors from repetitive statistical testing, multivariate techniques offer substantial advantages since they can take into account multiple factors that govern data variability simultaneously [51]. The arithmetic mean, standard deviation, minimum, and maximum are descriptive statistics of the soil characteristics being examined. SPSS version 25 was used for these calculations.

PCA condensed the dataset into new variables known as principle components (PCs) and eliminated multicollinearity between the original variables. These PCs account for most of the variation in the original variables. Principal components are linear combinations of variables that, if they are orthogonal to at least one another, represent vectors of closest fit for n observations in p-dimensional space [52]. This is by [53] PCs for an information set. For PCA in this work, 12 physical, chemical, and biological characteristics of the soil were used (Table 1). The quality of the land is sufficiently expressed by the soil qualities. Physical indicators tell us about root growth, plant emergence speed, and water infiltration, whereas chemical indicators tell us about organisms and nutrition availability, plant water, and contaminant movement [54]. The soil quality index and chemical soil parameters were found to be highly significantly correlated by Rangel-Peraza et al. [55].PCA’s goal was to reduce the amount of information while minimizing data loss [56]. The Kaiser–Meyer–Olkin (KMO) method assessed sample adequacy for the entire data set, with KMO values exceeding 0.5 signifying that the data is suitable for PCA. Moreover, the Bartlett test was used to assess data fitness, and the findings showed a p value of less than 0.05, which provided additional confirmation of the data’s suitability for PCA [17,51,57].Principal components (PC) with high eigenvalues larger than one were considered to be the finest representatives for comprehending the variability [37,58].

**Table 1:** Statistical analysis of selected soil properties.

Properties	Measuring units	N	Minimum	Maximum	Mean	STD
pH	–	51	7.62	8.44	8.01	0.22
EC	dS m^-1^	51	0.57	7.12	3.49	1.81
CaCO_3_	%	51	1.17	11.57	8.24	2.03
CEC	cmole kg^-1^	51	3.14	6.92	4.88	1
ESP	%	51	9.52	13.82	11.15	1.12
Bulk density	g cm^-3^	51	1.29	1.81	1.57	0.11
HC	cm h^-1^	51	40.69	216	106.76	38.49
OM	%	51	0.23	0.66	0.33	0.10
N	mg Kg^-1^	51	44.2	88.1	63.43	10.27
P	mg Kg^-1^	51	0.15	6.20	3.30	2.18
K	mg Kg^-1^	51	78.5	298.0	211.40	65.31
ECg	dS m^-1^	51	0.63	6.09	3.47	2.09

Note: HC = hydrlic conductivity, ECg = EC of ground water, N = number of samples, and STD = stander deviation.

### 2.4. The computation of the soil quality index (SQI)

The first stage, according to Mustafa [8] is to identify the minimal data sets (MDS) using PCA by Andrews [59] technique. The PCA under consideration is the principal component (PC) that accounts for at least 5% of the total cumulative variance and has an eigenvalue greater than 1. Second, each indicator underwent linear modifications to normalize all of its values to fall within the range of 0 and 1 [59]. Ultimately, the soil quality index (SQI) was calculated by combining the indicators:

Equation (1) was used to calculate the SQI by Cude [60], and Andrews [59].


$$SQI=\sum_{i=\text{1}}^{N}W_{i}\times S_{I}\;$$ (1)

where $S_{I}$ is the value of each soil indicator and $W_{i}$ is the relative weight of each indicator, which ranges from 0 to 1 [9,15].

The component score coefficient (CSC), which is derived from the PCA findings, is expressed by Wi. Equation (2) is used to standardize the Si values because the soil indicators have varying scales and units [61].


$$Z=X-\bar{\text{X}}÷\sigma$$ (2)

where Z, X, X and σ stand for the standardized value, the soil indicator’s value, the soil indicator’s average, and the soil indicator’s standard deviation, respectively [15,56,62].

Consequently, the principal components (PCs)-based SQI equation is as follows (Equation (3):


$$SQI-PC=\sum_{I=\text{1}}^{N}CSC\times z$$ (3)

Therefore, Equation (4) is used to calculate the comprehensive SQI (CSQI):


$$CSQI=\sum_{I=\text{1}}^{N}variability\;of\;each\;PC\times SQI-PC$$ (4)

Equation (5) converts the CSQI, which is determined using z scores, into a conventional normal distribution with a mean of 0 and a standard deviation of 1.


$$f\left(x\right)=1÷\sqrt{}2\pi {e^{-}}^{\frac{{\left(z\right)}^{2}}{2}}$$ (5)

Where **e** and **z** stand for the CSQI, which is calculated using z scores, and the natural logarithm, which is roughly equal to 2.718. The soil quality can be categorized as follows: very good (0–0.19), good (0.2–0.39), fair (0.4–0.59), bad (0.6–0.79), and extremely bad (0.8–1). Higher ratings indicate poorer conditions and worse land quality.

### 2.5. Geostatistical analyses

The ordinary Kriging (OK) method in ArcGIS10.8.1 was used to forecast the values of variables in unsampled locations using a geostatistical methodology [40,63–65]. Depending on their properties, the sophisticated geostatistical process known as (OK) can form a continuous surface from dispersed soil samples [66]. Using the average squared differences between each pair, semivariograms of the soil characteristics were produced (Equation 6) [67].


$$\gamma (h)=\text{1}÷\text{2}n(h)\sum_{i=\text{1}}^{N(h)}\left[Z(xi)-Z(XI+H)\right]\text{2}$$ (6)

**Fig 4:**
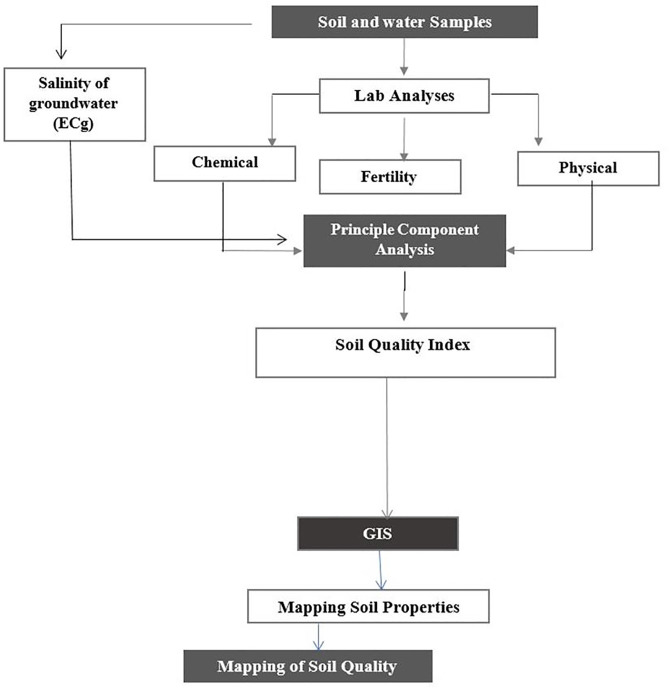
An outline of the steps involved in evaluating the quality of the soil.

Where Z(xi) is the measured sample value at point i, Z(xi + h) is the measured sample value at position (i + h), N(h) is the number of pairs of the lag interval, and g(h) is the semivariance of the distance interval h. The semivariogram is a statistic that can be used in exploratory data analysis since it evaluates the average decline in similarity between two random variables as the distance between the variables grows [68]. The nugget effect, range, and sill are the three primary characteristics that characterize the semivariogram in general. The nugget effect is a discontinuity of the variogram that expresses non-spatial variation as well as variability at a scale smaller than the sampling interval. The nugget effect, which cannot be eliminated by close sampling, can only be eliminated by repeated measurements [69]. For uncorrelated samples, range and sill represent lag distance and distance, respectively [70]. Mean error (ME), root-mean-square error (RMSE), mean standardized error (MSE), and root-mean-square standardized error (RMSSE), were used to choose the best semivariogram models. If the RMSEE is close to one and the ME and MSE values are close to zero, the forecasting model is of exceptional quality and applicability [37,69,71,72]. Moreover, a nugget –to- sill (SDC) ratios of less than 0.25, 0.25–0.75, and greater than 0.75 suggest significant, moderate, and weak spatial dependence, respectively [73], Fig 4 displays the flowchart of the steps taken in this investigation to calculate the soil quality index.

## 3. Results and discussion

### 3.1. Soil characteristics and geomorphological units of the of the study area

**Fig 5:**
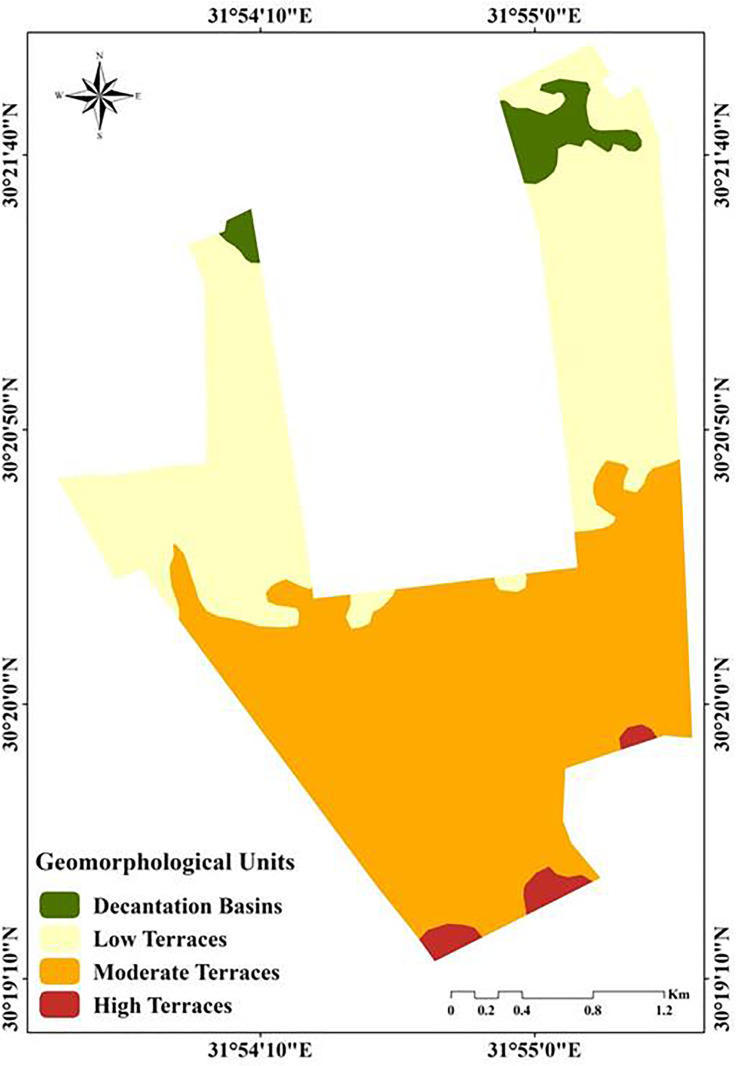
Geomorphological units of the study area.

The main geomorphological units in the area are decantation basins, low, moderate, and high terraces (Fig 5), with areas of 20.23, 249, 335.31, and 9.78 ha, respectively.

**Fig 6:**
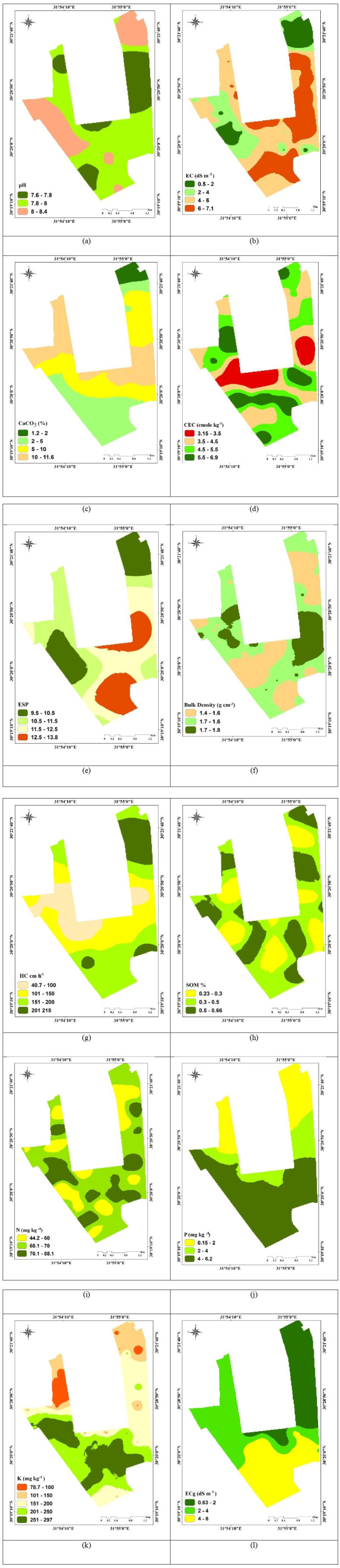
The spatial distribution maps of the soil parameters in the study area (a) pH, (b) EC(dS m^-1^), (c) CaCO_3_(%), (d) CEC (cmole_c_ Kg^-1^), (e) ESP, (f) bulk density(g cm^-3^), (g) HC (cm h^-1^), (h) SOM%, (i) N (mg Kg^-1^), (j) P(mg Kg^-1^),(k) k (mg kg^-1^), and(l) ECg(dSm^-1^).

The average pH value is 8.01 ± 0.22, with a range of 7.62 to 8.44 (Table 1 and Fig 6a). All samples were alkaline soils with a pH of 7.0 or above. A pH greater than 8 suggests that there may be substantial amounts of exchangeable calcium or magnesium present. The different observed pH values for the soils under study show that the soil is alkaline, which could be explained by carbonate hydrolysis or a lack of precipitation [74,75].

The findings demonstrated that the electrical conductivity of the majority of the farm’s samples varied with salinity and that depth and texture are significant determinants of agricultural priorities. In this area, salinity is regarded as the most significant factor due to the use of contemporary irrigation, but it is not a serious issue because irrigation with fresh water does not exceed 1000 parts per million. The EC values in the studied area range from 0.57 to 7.12 dSm^-1^, with an average of 3.49 ± 1.81 dSm^-1^ (Table 1 and Fig 6b), indicating none to high salinity soils. Because of low precipitation and high evaporation, the majority of salinized soils are found in arid and semi-arid environments [76]. Along with the presence of gravel in varying amounts at various distances from the sector (i.e., the soil is easily washed and salt-free and salts are kept away from the root spread zone). The average CaCO_3_ is 8.24 ± 2.03%, with a range of 1.17 to 11.57% (Table 1 and Fig 6c). The formation of very hard, water-impermeable layers and crops can occur in areas with the greatest CaCO_3_ values [77]. The average CEC result was 4.88 ± 1%, with a range of 0.63 to 6.09% (Table 1 and Fig 6d). Since there are strong positive relationships between CEC, clay, and organic matter, the low values of it are caused by low levels of both [15]. The area does not confront sodicity dangers, as indicated by the ESP values, which vary from 9.52 to 13.82 with an average of 11.15 ± 1.12 (Table 1 and Fig 6e) [78],.The bulk density (Bd) ranged from 1.29–1.81 G cm^-3^ Table 1 and Fig 6f).

The average soil organic matter (SOM) content is 0.33 ± 0.10, with a range of 0.23 to 0.66 (Table 1 and Fig 6g). It was determined that the research area’s soils had a low SOM% content [49,79]. SOM %is essential for enhancing the physical and chemical characteristics of soil [80,81].

According to Abdellatif et al., [82] and Semih and Kenan [83], hydraulic conductivity is a primary indicator of soil pore structure and water movement. (HC) averages 106.76 ± 38.49, with a range of 40.69 to 216. (Table 1 and Fig 6h) The available N varies between 44.2 and 88.1 mg kg^-1^, indicating that the study area’s nitrogen content varies from low to moderate [49], with certain areas having a high N content as a result of farming activities (Table 1 and Fig 6i). According to [49], the research area’s available P and K content is categorized as low to moderate since the average values are 3.3 and 11.4 mg kg^-1^, respectively (Table 1 and Fig 6j, k). The average ground water salinity (ECg) in the studied area was 3.47 dS m^-1^, with values ranging from 0.63 to 6.09 dS m^-1^ (Table 1 and Fig 6l).

### 3.2. Studied variables mapping

The unknown values of soil parameters were estimated and mapped using the ordinary Kriging interpolation method. As indicated in Table 2, the accuracy of the model was validated for every soil property using ME, RMSE, MSE, and RMSSE. The findings indicate that the J-Bessel model for ESP, SOM, and N, and the exponential model for the (CaCO_3_, HC, and P) are most suited for forecasting unknown values. The best model for EC and BD is stable. For pH, CEC, ECg, and K, respectively tetra spherical, penta spherical, circular, and K-Bessel are appropriate. Furthermore, the findings show that for the chosen soil parameters, the MSE is around zero and the RMSSE is near one; consequently, the chosen models are appropriate for forecasting the unsampled soil parameters and fit the data [38,84]. Between 580.6 m (N) and 4112.7 m (P), the geostatistical range values for the soil parameters were clearly found. The broad range value suggests that some other values of this variable have bigger effects on the observed values of the soil property over longer distances than soil variables with narrower ranges (Table 2). Spatial dependence varies from mild to considerable for soil properties. Orthic factors like fertilization and cultivation techniques create weak spatial dependence, whereas both hereditary and orthic factors impact moderate spatial dependence [29,73,85]. Sites located in the northeastern and southeastern parts of the study area exhibited the highest pH values (around 8–8.4) (Fig 6a). The values of EC, CaCO_3_%, CEC, and ESP rose from the northern to the southern part of the study area (Fig 6b–e). Throughout the research area, SOM levels are low, but they are particularly lowest in certain areas of the middle and southern parts of the study area (Fig 6h). As illustrated in Fig 6j and k, the concentrations of both P and K exhibited an increase from the northeast to the south throughout the study area.The spatial interpolation of ECg exhibited an upward trend from the northeast to the south of the study area (Fig 6i)

**Table 2:** Geostatistical models characteristics of the studied variables.

Variable	Model	Measuring unit	Nugget	Partial	Sill	Nugget/Sill	Major	SDC	Me	RMES	MSE	RMSSE
			(C0)	sill	(C0 + C)		Range					
pH	Tetra spherical	–	0.44	0.42	0.86	0.51	1140.8	Moderate	0.004	0.18	0.028	1.01
EC	Stable	dS m^-1^	0.44	0.56	1	0.44	608.9	Moderate	−0.03	1.74	−0.01	1.04
CaCO_3_	Exponential	%	0.53	0.64	1.17	0.45	3424.9	Moderate	0.064	1.96	0.041	1.03
CEC	Penta spherical	cmole kg^-1^	0.089	1.03	1.12	0.079	798.7	Strong	0.02	0.93	0.012	1.03
ESP	J-Bessel	%	0.79	0.17	0.96	0.82	1360.9	Weak	−0.01	1.08	−0.01	1.03
Bulk Density	Stable	g cm^-3^	0	0.91	0.91	0	2111.8	Strong	0.01	1.2	0.035	1.23
HC	Exponential	cm h^-1^	0.65	0.84	1.49	0.44	2948.6	Moderate	−1.7	36.19	−0.05	1.06
SOM	J-Bessel	%	0.86	0.11	0.97	0.88	903.02	Weak	0	0.09	−0.04	0.99
N	J-Bessel	mg Kg^-1^	0.21	0.81	1.02	0.2	580.6	Strong	−0.19	10.14	−0.02	1.05
P	Exponential	mg Kg^-1^	0.55	0.69	1.24	0.44	4112.7	Moderate	0.07	1.8	0.03	0.99
K	K-Bessel	mg Kg^-1^	0	1.06	1.06	0	785.5	Strong	1.73	61.6	0.029	0.99
ECg	Circular	dS m^-1^	0	1.1	1.1	0	2111.8	Strong	0.01	1.2	0.035	1.23

### 3.3. Principal component analysis for the soil quality index

According to the findings, the sample size is appropriate for evaluating the factor structure because the KMO value is higher than 0.5 [17,51], and It also revealed a p < 0.01, which further confirmed the data’s suitability for PCA [86] (S1 Table in S1 File). A Principal Component Analysis is acceptable as the variables in the model may explain the phenomena and the test results show that the variables are not entirely uncorrelated [15,87–89]. Table 3 displays the PCA results. According to the approach suggested by Kaiser (1960), the top five Principal Components (PCs) were used since their eigenvalues were greater than 1, and the remaining PCs were rejected (Tables 4, 5 and S2 in S1 File). The results show that the first five PCs account for 72.89% of the variance. The first PC, which accounts for 23.361% of the total variation, has stronger relationships with pH, EC, and ESP, whereas the second PC, which accounts for 18.306% of the variance, has positive correlations with ECg, SOM, and available N, P, and K, according to the factor loadings. The third PC is associated with Bd, and HC and accounts for 12.140% of the overall variance. 10.48% of the variance is explained by the fourth PC and is associated with CEC. Finally the fifth PC 8.60% of the variance and correlated with CaCO_3_%. Based on the PCA results, the soil quality index was developed using equations (7–11).

**Table 3:** Summarization of Principle component analysis of the studied variables.

		PC1	PC2	PC3	PC4	PC5
Eigenvalue		2.803	2.19	1.45	1.25	1.03
Variability (%)		23.361	18.30	12.14	10.48	8.60
Cumulative %		23.361	41.66	53.80	64.29	72.89
pH	PC loading	−.704	.198	.193	.242	−.074
EC		.938	−.035	−.107	.069	.122
CaCO_3_		.307	−.089	.330	−.174	.713
Ecg		.123	.695	.097	.449	.165
CEC		−.030	.181	.142	.704	.011
ESP		.928	.032	−.144	.076	.026
Bd		.331	−.190	.712	.158	−.284
HC		−.288	.374	−.639	−.094	.228
OM		0.244	0.478	.312	−.086	−.558
N		−.038	.522	.387	−.511	.007
P		.149	.808	−.062	.133	.083
K		.060	.686	.288	−.375	−.159
pH	Component Score Coefficient (CSC) matrix	−0.251	0.090	0.133	0.193	−0.072
EC		0.334	−0.016	−0.074	0.055	0.118
CaCO_3_		0.109	−0.041	0.226	−0.138	0.691
Ecg		0.044	0.316	0.067	0.357	0.160
CEC		−0.011	0.082	0.097	0.560	0.011
ESP		0.331	0.014	−0.099	0.061	0.025
Bd		0.118	−0.087	0.489	0.125	−0.275
HC		−0.103	0.170	−0.439	−0.075	0.221
OM		0.174	0.111	−0.214	−0.069	−0.540
N		−0.014	0.238	0.266	−0.406	0.007
P		0.053	0.368	−0.042	0.106	0.081
K		0.021	0.312	0.198	−0.298	−0.154

**Table 4:** Normalized Z score of the variables which used in the soil quality.

Normalized Z score											
pH	EC	CaCO_3_	CEC	ESP	Bulk density	HC	SOM	N	P	K	ECg
0.49	0.13	0.01	0.76	0.10	0.46	0.45	0.16	0.50	0.07	0.21	0.00
1.00	0.01	0.00	0.43	0.00	0.46	0.44	0.51	0.34	0.11	0.54	0.00
0.65	0.10	0.46	0.40	0.07	0.42	0.58	0.00	0.17	0.06	0.27	0.00
0.84	0.00	0.71	0.40	0.01	0.42	0.58	0.05	0.17	0.06	0.02	0.00
0.75	0.03	1.00	0.49	0.03	0.42	0.65	0.00	0.17	0.30	0.96	0.00
0.19	0.57	0.95	0.42	0.42	0.44	0.47	0.33	0.21	0.00	0.22	0.54
0.02	1.00	0.39	0.29	0.71	0.44	0.55	0.51	0.21	0.00	0.56	0.00
0.00	0.64	0.88	0.52	0.45	0.42	0.65	0.10	0.73	0.45	0.61	0.00
0.43	0.50	0.81	0.92	0.37	0.44	0.49	0.10	0.73	0.45	0.00	0.54
0.20	0.38	0.67	0.53	0.27	0.77	0.23	0.44	0.17	0.06	0.45	0.00
0.05	0.71	0.74	0.40	0.56	0.17	0.56	0.44	0.50	0.95	0.81	0.00
0.49	0.14	0.58	0.70	0.11	0.81	0.03	0.05	0.21	0.00	0.04	0.54
0.31	0.59	0.92	0.61	0.49	0.81	0.12	0.00	0.34	0.11	0.10	0.54
0.21	0.62	0.69	0.02	0.48	0.81	0.08	0.09	0.37	0.12	0.03	0.00
0.03	0.69	0.75	0.27	0.59	0.81	0.06	0.00	0.36	0.12	0.52	0.00
0.76	0.52	0.79	0.37	0.55	0.81	0.03	0.44	0.50	0.90	0.81	0.54
0.64	0.30	0.92	0.48	0.14	0.42	0.02	0.00	0.36	0.12	0.52	0.54
0.83	0.10	0.60	0.58	0.08	0.85	0.45	0.02	0.73	0.45	1.00	0.54
0.46	0.66	0.82	0.94	0.56	0.62	0.06	0.00	0.36	0.12	0.52	0.54
0.43	0.66	0.96	0.19	0.52	1.00	0.44	0.10	1.00	0.90	0.53	0.00
0.74	0.03	0.55	0.16	0.02	0.46	0.45	0.02	1.00	0.90	0.45	0.54
0.69	0.16	0.64	0.11	0.13	0.81	0.03	0.02	0.73	0.45	1.00	0.54
0.33	0.49	0.62	0.05	0.38	0.42	0.44	0.44	0.50	0.95	0.81	0.54
0.56	0.16	0.84	0.18	0.07	0.42	0.06	0.05	0.73	0.45	1.00	0.54
0.13	0.92	0.92	0.03	1.00	0.81	0.00	0.44	0.00	0.00	0.00	0.00
0.34	0.48	0.59	0.06	0.37	0.46	0.39	0.44	0.50	0.95	0.80	1.00
0.20	0.64	0.72	0.00	0.49	0.00	0.39	0.09	0.37	0.12	0.00	0.00
0.78	0.27	0.95	0.39	0.21	0.46	0.39	0.55	0.59	0.29	1.00	0.00
0.41	0.70	0.59	0.89	0.68	0.92	0.38	0.44	0.50	0.95	0.79	0.81
0.48	0.82	0.84	0.81	0.29	0.81	0.37	0.00	0.27	0.67	0.01	0.81
0.79	0.10	0.59	0.47	0.10	0.42	0.32	0.00	0.02	0.83	0.41	0.54
0.72	0.23	0.59	0.97	0.21	0.42	0.32	0.44	0.26	1.00	0.43	0.54
0.73	0.19	0.80	0.31	0.15	0.42	0.25	0.51	0.36	0.74	0.68	1.00
0.40	0.55	0.69	0.95	0.48	0.42	0.26	0.10	0.73	0.45	1.00	1.00
0.29	0.96	0.68	1.00	0.92	0.73	0.45	0.53	0.52	0.79	0.83	0.81
0.63	0.07	0.58	0.72	0.06	0.46	0.45	0.07	0.73	0.45	1.00	0.81
0.21	0.62	0.64	0.31	0.62	0.75	0.10	1.00	0.52	0.79	0.41	0.81
0.65	0.05	0.63	0.33	0.05	0.33	1.00	0.07	0.73	0.45	1.00	0.81
0.26	0.38	0.51	0.31	0.28	0.33	1.00	0.12	0.27	0.82	0.36	1.00
0.17	0.71	0.63	0.38	0.52	0.69	0.44	0.10	0.73	0.45	1.00	1.00
0.87	0.84	0.60	0.36	0.78	0.69	0.44	0.00	0.02	0.83	0.41	1.00
0.34	0.66	0.76	0.54	0.66	0.12	0.44	0.51	0.36	0.74	0.68	1.00
0.21	0.70	0.57	0.39	0.51	0.50	0.44	0.10	0.73	0.45	1.00	1.00
0.27	0.37	0.57	0.77	0.27	0.50	0.44	0.12	0.27	0.82	0.36	1.00
0.68	0.40	0.71	0.48	0.38	0.42	0.44	0.07	0.36	0.90	0.18	1.00
0.73	0.49	0.58	0.53	0.41	0.44	0.44	0.07	0.36	0.90	0.18	1.00
0.40	0.67	0.60	0.46	0.70	0.62	0.44	0.51	0.36	0.74	0.68	1.00
0.93	0.18	0.86	0.39	0.14	0.23	0.44	0.00	0.26	0.74	0.00	0.54
0.55	0.53	0.75	0.28	0.86	0.25	0.44	0.53	0.41	0.79	0.00	0.54
0.29	0.51	0.73	0.68	0.42	0.42	0.44	0.53	0.52	0.79	0.18	0.54
0.80	0.57	0.72	0.43	0.64	0.42	0.44	0.44	0.50	0.90	0.00	0.54

**Table 5:** Comprehensive soil quality index (CSQI) of the study area.

SQI-PC1	SQI-PC2	SQI-PC3	SQI-PC4	SQI-PC5	CSQI
−0.016	0.372	0.289	0.286	−0.156	0.12
−0.142	0.510	0.288	0.133	−0.445	0.07
−0.067	0.282	0.262	0.164	0.251	0.10
−0.134	0.215	0.300	0.226	0.423	0.10
−0.048	0.592	0.511	−0.034	0.549	0.20
0.466	0.378	0.273	0.238	0.516	0.27
0.698	0.342	0.008	−0.063	−0.075	0.21
0.479	0.619	0.351	−0.199	0.543	0.29
0.319	0.644	0.447	0.494	0.604	0.34
0.387	0.254	0.488	0.132	−0.004	0.21
0.587	0.856	0.100	−0.188	0.319	0.31
0.140	0.250	0.716	0.611	0.217	0.24
0.486	0.313	0.699	0.460	0.517	0.34
0.495	0.085	0.531	−0.038	0.223	0.21
0.599	0.226	0.655	−0.065	0.259	0.27
0.498	0.922	0.803	0.216	0.093	0.41
0.164	0.487	0.731	0.242	0.507	0.28
0.028	0.925	0.908	0.132	0.193	0.31
0.475	0.503	0.739	0.548	0.422	0.38
0.521	0.747	0.799	−0.258	0.410	0.36
−0.027	0.973	0.610	−0.071	0.387	0.27
0.146	0.804	1.008	−0.130	0.138	0.30
0.439	0.986	0.319	−0.104	0.197	0.33
0.135	0.830	0.841	−0.203	0.385	0.30
0.879	−0.044	0.362	0.099	0.188	0.26
0.460	1.120	0.388	0.079	0.232	0.38
0.380	0.193	−0.005	−0.167	0.540	0.15
0.204	0.709	0.593	−0.251	0.146	0.24
0.658	1.091	0.649	0.583	0.085	0.49
0.461	0.628	0.573	0.810	0.607	0.42
0.021	0.712	0.422	0.525	0.402	0.27
0.197	0.920	0.406	0.687	0.191	0.35
0.217	1.006	0.532	0.306	0.284	0.35
0.429	1.071	0.722	0.409	0.400	0.45
0.836	1.078	0.498	0.564	0.159	0.52
0.034	1.043	0.714	0.213	0.317	0.33
0.759	0.877	0.445	0.254	−0.144	0.40
−0.042	1.117	0.383	−0.068	0.506	0.27
0.256	0.975	−0.004	0.375	0.586	0.32
0.564	1.013	0.660	0.089	0.336	0.43
0.517	0.853	0.411	0.750	0.448	0.44
0.582	1.050	0.176	0.370	0.429	0.42
0.521	1.036	0.559	0.086	0.345	0.41
0.325	0.901	0.389	0.690	0.461	0.39
0.269	0.908	0.391	0.611	0.604	0.38
0.282	0.920	0.374	0.679	0.507	0.39
0.624	1.012	0.381	0.426	0.178	0.43
0.006	0.636	0.323	0.456	0.716	0.26
0.540	0.715	0.070	0.314	0.391	0.33
0.470	0.787	0.268	0.384	0.320	0.35
0.423	0.785	0.258	0.437	0.365	0.35


$$\begin{array}{cc} SQI-PC\text{1}= & -\text{0}.\text{2}\text{5}\text{1}\times ZpH+\text{0}.\text{3}\text{3}\text{4}\times ZEC+\text{0}.\text{1}\text{0}\text{9}\times ZCaCO\text{3}-\text{0}.\text{0}\text{1}\text{1}\times ZCEC\times \text{0}.\text{3}\text{3}\text{1}\times ZESP\\ & +\text{0}.\text{1}\text{1}\text{8}\times ZBD-\text{0}.\text{1}\text{0}\text{3}\times ZHC+\text{0}.\text{1}\text{7}\text{4}\times ZSOM\pm \text{0}.\text{0}\text{1}\times ZN+\text{0}.\text{0}\text{5}\times ZP+\text{0}.\text{0}\text{2}\times ZK\\ & +\text{0}.\text{0}\text{4}\times ZECg \end{array}$$ (7)

$$\begin{array}{cc} SQI-PC\text{2}= & \;\text{0}.\text{0}\text{9}\times ZpH-\text{0}.\text{0}\text{1}\text{6}\times ZEC-\text{0}.\text{0}\text{4}\times ZCaCO\text{3}+\text{0}.\text{0}\text{8}\text{2}\times ZCEC+\text{0}.\text{0}\text{1}\text{4}\times ZESP\\ & -\text{0}.\text{0}\text{8}\text{7}\times ZBD+\text{0}.\text{1}\text{7}\text{0}*ZHC+\text{0}.\text{1}\text{1}\text{1}\times ZSOM+\text{0}.\text{2}\text{3}\text{8}\times ZN+\text{0}.\text{3}\text{6}\text{8}\times ZP\\ & +\text{0}.\text{3}\text{1}\text{2}\times ZK+\text{0}.\text{3}\text{1}\text{6}\times ZECg \end{array}$$ (8)

$$\begin{array}{cc} SQI-PC\text{3}= & \;\text{0}.\text{1}\text{3}\text{3}\times ZpH-\text{0}.\text{0}\text{7}\text{4}\times ZEC+\text{0}.\text{2}\text{2}\text{6}\times ZCaCO\text{3}+\text{0}.\text{0}\text{9}\text{7}\times ZCEC-\text{0}.\text{0}\text{9}\text{9}\times ZESP\\ & +\text{0}.\text{4}\text{8}\text{9}\times ZBD-\text{0}.\text{4}\text{3}\text{9}\times ZHC-\text{0}.\text{2}\text{1}\text{4}\times ZSOM+\text{0}.\text{2}\text{6}\text{6}\times ZN-\text{0}.\text{0}\text{4}\text{2}\times ZP\\ & +\text{0}.\text{1}\text{9}\text{8}\times ZK+\text{0}.\text{0}\text{6}\text{7}\times ZECg \end{array}$$ (9)

$$\begin{array}{cc} SQI-PC\text{4}= & \;\text{0}.\text{1}\text{9}\text{3}\times ZpH+\text{0}.\text{0}\text{5}\text{5}\times ZEC-\text{0}.\text{1}\text{3}\text{8}\times ZCaCO\text{3}+\text{0}.\text{5}\text{6}\text{0}\times ZCEC+\text{0}.\text{0}\text{6}\text{1}\times ZESP\\ & +\text{0}.\text{1}\text{2}\text{5}\times ZBD-\text{0}.\text{0}\text{7}\times ZHC-\text{0}.\text{0}\text{6}\text{9}\times ZSOM-\text{0}.\text{4}\text{0}\text{6}\times ZN+\text{0}.\text{1}\text{0}\text{6}\times ZP\\ & -\text{0}.\text{2}\text{9}\text{8}\times ZK+\text{0}.\text{3}\text{5}\text{7}\times ZECg \end{array}$$ (10)

$$\begin{array}{cc} SQI-PC\text{5}= & -\text{0}.\text{0}\text{7}\times ZpH+\text{0}.\text{1}\text{1}\text{8}\times ZEC+\text{0}.\text{6}\text{9}\text{1}\times ZCaCO\text{3}+\text{0}.\text{0}\text{1}\text{1}\times ZCEC+\text{0}.\text{0}\text{1}\times ZCEC\\ & +\text{0}.\text{0}\text{2}\text{5}\times ZESP-\text{0}.\text{2}\text{7}\text{5}\times ZBD+\text{0}.\text{2}\text{2}\text{1}\times HC-\text{0}.\text{5}\text{4}\text{0}\times SOM+\text{0}.\text{0}\text{0}\text{7}\times ZN\\ & +\text{0}.\text{0}\text{8}\text{1}\times ZP-\text{0}.\text{1}\text{5}\text{4}\times ZK+\text{0}.\text{1}\text{6}\text{0}\times ZECg \end{array}$$ (11)

### 3.4. Mapping soil quality index

**Fig 7:**
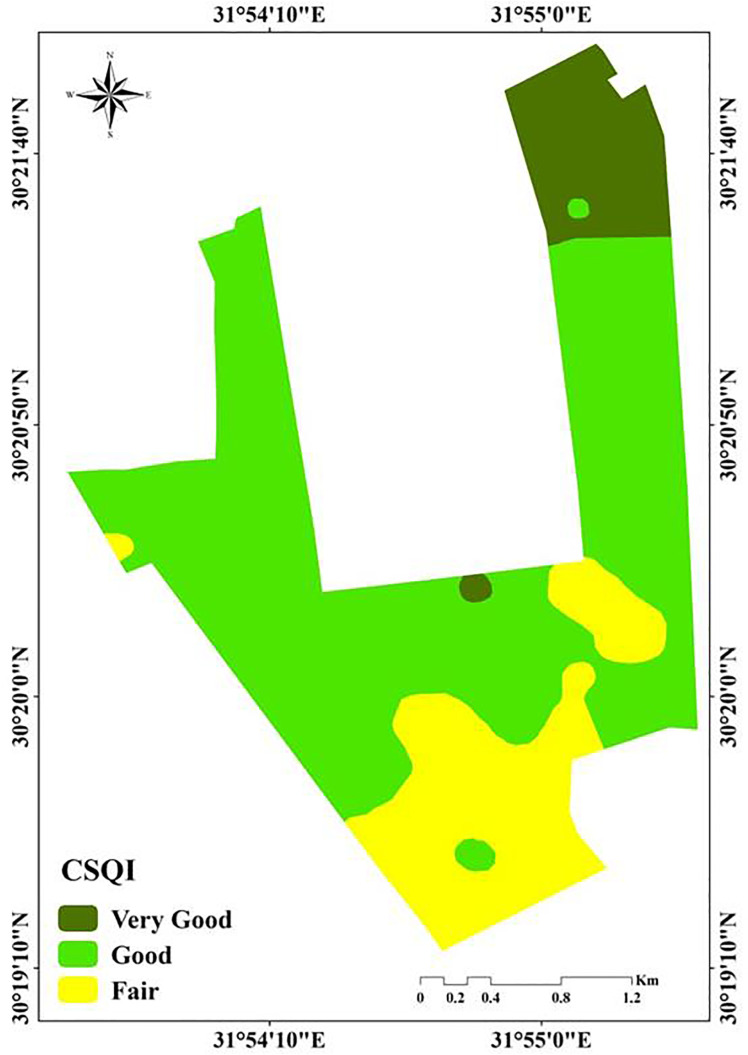
Soil quality distribution within the study area.

Based on the CSQI values, which were computed using Equation (4), OK interpolation was utilized to interpolate the spatial variability of soil quality in the research region. Table 6 and Fig 7 display the findings, the SQI’s findings fall between 0.07 and 0.52. As seen in the Fig 7 and Table 6, the SQI is divided into three quality zones. The composition of the soil and the surrounding climate and environment have an impact on it [84,90,91]. The lowest restrictions were found in the northeastern zone. These soils were non-sodic (ESP average 10), moderately calcareous (average CaCO_3_ 5%), non-saline (average 1.7 dS m^-1^ and groundwater; average 0.63 dS m^−1^) (Fig 7). Good soil quality is a defining feature of the second zone, which makes up roughly 414.76 ha (67.5%) of the entire region located in the middle part of the study area (Fig 7). The third zone, which makes up around 133.91 ha (21.8%) of the entire land, is fair (poor quality) south of the study area. In the second zone, groundwater and soil salinity were moderate (3.24, and3.27, respectively, whereas in the third zone, they were high (5.73, and 5.37 dS m^-1^). Furthermore, the third zone’s ESP (12.17%) was higher than the second zone’s (10.9%) and the average of bd was high (1.6 g cm^-3^) according to [92]. Our findings were consistent with those of Yousif et al., [40] who divided the study area into three zones and demonstrated that the area under investigation included three site-specific management zones (SSMZs). The second site zone, which denotes areas with low productivity, includes the majority of the studied area. The most efficient factors influencing the SQI in the research region were organic matter, ECe, ECg, ESP, and Bd. The SQI is negatively impacted by these factors’ low values [40]. Physical indicators such as bulk density, porosity, aggregate stability, texture, and compaction influence how the particles and pores are arranged, which explains how they affect water infiltration, root growth, and plant emergence speed. Based on studies carried out in comparable dry conditions, long-term management and restoration efforts of the third zone may progressively enhance soil quality [93,94]. To make the most use of the resources available, less demanding crops or alternative land uses like grazing or agroforestry might be grown in this third zone. With minimal outside assistance, farmers in Zone one can concentrate on high-value crops and carry out intensive agricultural activities.

**Table 6:** Areas and classes of SQI in the study area.

Classes	Hectare	%
Very Good	65.66	10.7
Good	414.76	67.5
Fair	133.91	21.8

However, to maintain sustainability, some precision farming interventions will be required for efficient management in this area. Oliver et al. [95] state that similar research emphasizes the importance of these elements in promoting sustainable agricultural development. According to research on precision farming, areas with superior soil qualities typically have higher yields and lower input needs [96,97], As stated by Oliver et al. [95], management strategies in this area should give top priority to preserving soil fertility, avoiding excessive fertilization, and encouraging sustainable intensification. Even though the soils were suitable in zone 2 for farming, further management techniques like variable-rate fertilization and customized irrigation schedules will be needed to optimize production. The soil parameters in this zone showed regional variation, indicating the potential utility of precision farming techniques [98,99]. Although this zone is good for agriculture, it might be essential to carefully regulate inputs like fertilizer and targeted irrigation to control the fluctuation in soil qualities. In regions with moderate soil quality, variable-rate approaches were employed to maximize resource utilization; other soil management studies have identified comparable zones [99,100]. Effective management in this area may guarantee sustainable productivity with lower input costs.

Zone three may need the biggest financial investment for plans including alternative land uses or soil restoration because of its unfavorable features [40]. By leveraging crop functions, root systems, and nutrient utilization, as well as by fostering a diversified microbial community, crop rotation may aid in promoting soil health. This diversity benefits the soil by improving nutrient cycling and fertility, reducing erosion, increasing carbon sequestration, and decreasing compaction [101]. According to studies done under comparable dry conditions, long-term management and restoration approaches may progressively enhance soil quality [91,[93,94]. This zone could be utilized for less demanding crops or other land uses, such as agroforestry or grazing, to maximize the utilization of the resources available. Although PCA is a popular technique for determining soil quality, it occasionally falls short of accurately differentiating soil quality [13]. As the primary drawback of statistical approaches is their incapacity to incorporate nonlinear interactions and the spatial dimension of the variables under study [102]. The enhancement does not depend on statistical techniques; rather, extensive soil variable data should be utilized to enhance the GIS geostatistical models’ prediction process [103].

## 4. Conclusions

Accurately assessing the quality of the soil is crucial for both sustainable agriculture management (in general) and precision farming (in specific). This assessment makes it easier to determine the best crops to grow there as well as the area’s possible agricultural applications. Soil quality is influenced by weather patterns and agricultural practices, which in turn affect the fertility, chemical, and physical properties of the soil. SQI in the southwest of Egypt’s Ismailia Governorate was evaluated in this study using the nutrients and the physical and chemical characteristics of the soil. The use of GIS to map soil properties immediately highlighted the differences and geographical variations in SQI from one area to another. The results show that the exponential model for (CaCO_3_, HC, and P) and the J-Bessel model for ESP, SOM, and N are most suited for predicting unknown values. Stable is the ideal model for BD and EC. Tetra spherical, penta spherical, circular, and K-Bessel are suitable for pH, CEC, ECg, and K, respectively. Additionally, the results demonstrate that the MSE is close to zero and the RMSSE is close to one for the selected soil characteristics; as a result, the models selected are suitable for predicting the unsampled soil parameters and fit the data. The SQI is divided into three quality zones. The first zone, which makes up roughly 65.66 (ha) of the total area, has a very good quality index. This zone’s soils are characterized by low groundwater salinity and sufficient levels of each soil characteristic. The second zone, which makes up about 414.76 ha (67.5%) of the entire region, is distinguished by its good soil quality. About 21.8% of the land area, or 133.91 hectares, is in the third zone, which is classified as fair (poor quality). Low concentrations of SOM, salinity, available N, P, K, and CEC had the greatest effects on the SQI of the studied location. Overall, the findings demonstrate that a precise and efficient evaluation of the SQI is possible through the combined application of PCA and GIS. The identification of soil quality zones has resulted in an effective agricultural planning tool for the research region. By adapting farming practices to the particular needs of each zone, resource consumption efficiency can be maximized, reducing input costs and environmental effects. With minimal outside assistance, farmers in Zone one are able to concentrate on high-value crops and carry out intensive agricultural activities. In Zone two, more effective resource management will be made possible by precision agricultural methods. Zone three may demand the highest financial investment for ideas requiring alternative land uses or soil remediation because of its unfavorable qualities. From an environmental perspective, management plans based on soil quality zoning lower the danger of water runoff and soil deterioration by preventing excessive use of water and pesticides. This supports environmental health and is in line with sustainable farming methods. Without using general procedures across the research area, one can increase overall farm productivity and maximize yield in high-potential areas by concentrating on necessary intense techniques. This research emphasizes the crucial role of GIS and PCA in soil quality monitoring, aiding decision-makers in implementing effective improvement strategies aligned with the Sustainable Development Goals (SDGs).

As suggested by the researchers, a thorough shift will take place, resulting in the steady advancement of artificial intelligence (AI) applications in agriculture via a number of advanced strategies. With its many benefits, artificial intelligence (AI) has revolutionized the agricultural food production process. For predicting soil quality, machine learning (ML) and deep learning (DL) hold great promise for accuracy and efficacy. Because machine learning and deep learning can forecast the soil quality index without the need for more intricate calculations, this makes their application special.

## Supporting information

S1 File Supplementary data. (DOCX)
