# Supplementary material for: Assessment of soil quality in arid zones using principal component analysis and GIS-based modeling
Source: PLoS One. 2025 Dec 2;20(12):e0337063. doi: 10.1371/journal.pone.0337063 (PMC12671789; doi:10.1371/journal.pone.0337063)
Supplement: S1 File — (DOCX) [file pone.0337063.s001.docx]

Table S1 KMO and Bartlett's test of the studied variables

| **KMO and Bartlett's Test** | | |
| --- | --- | --- |
| Kaiser-Meyer-Olkin Measure of Sampling Adequacy. | | 0.546 |
| Bartlett's Test of Sphericity | Approx. Chi-Square | 217.498 |
|  | df | 66 |
|  | Sig. | 0.000 |

Table S2 all PC componentS resulted from PCA

| **Total Variance Explained** | | | | | | |
| --- | --- | --- | --- | --- | --- | --- |
| Component | Initial Eigenvalues | | | Extraction Sums of Squared Loadings | | |
|  | Total | % of Variance | Cumulative % | Total | % of Variance | Cumulative % |
| 1 | 2.803 | 23.361 | 23.361 | 2.803 | 23.361 | 23.361 |
| 2 | 2.197 | 18.306 | 41.668 | 2.197 | 18.306 | 41.668 |
| 3 | 1.457 | 12.140 | 53.808 | 1.457 | 12.140 | 53.808 |
| 4 | 1.258 | 10.483 | 64.291 | 1.258 | 10.483 | 64.291 |
| 5 | 1.033 | 8.605 | 72.896 | 1.033 | 8.605 | 72.896 |
| 6 | 0.978 | 8.151 | 81.047 |  |  |  |
| 7 | 0.689 | 5.744 | 86.792 |  |  |  |
| 8 | 0.521 | 4.339 | 91.131 |  |  |  |
| 9 | 0.444 | 3.698 | 94.829 |  |  |  |
| 10 | 0.323 | 2.690 | 97.519 |  |  |  |
| 11 | 0.240 | 2.002 | 99.520 |  |  |  |
| 12 | 0.058 | 0.480 | 100.000 |  |  |  |
| Extraction Method: Principal Component Analysis. | | | | | | |
